# Connective tissue profiling in keratinized and non-keratinized oral mucosa reveals distinct extracellular and intracellular features

**DOI:** 10.3389/fcell.2026.1815527

**Published:** 2026-06-12

**Authors:** Ruoxuan Huang, Leyao Xu, Chunhsin Hsu, Yuanxiang Liu, Runheng Liu, Shudan Deng, Zhipeng Li, Zetao Chen, Shoucheng Chen, Zhuofan Chen

**Affiliations:** Hospital of Stomatology, Guanghua School of Stomatology, Sun Yat-sen University and Guangdong Provincial Key Laboratory of Stomatology and Guangdong Research Center for Dental and Cranial Rehabilitation and Material Engineering, Guangzhou, China

**Keywords:** connective tissue, extracellular matrix remodeling, immune microenvironment, oral mucosal keratinization, retinol metabolism

## Abstract

**Introduction:**

Keratinized mucosa is a critical determinant of long-term periodontal and peri-implant tissue stability. Emerging evidence suggests that the underlying connective tissue plays a pivotal role in defining oral mucosal phenotypes. However, the mechanisms by which it shapes the mucosal microenvironment and regulates epithelial keratinization remain incompletely understood. This study aimed to map the molecular blueprints of in situ connective tissue from human keratinized (gingiva, GIN) and non-keratinized (alveolar mucosa, ALV) oral mucosa.

**Methods:**

Connective tissue samples were harvested and analyzed by transcriptomic sequencing, qPCR, IHC, WB and IF.

**Results:**

Differential analysis revealed distinct extracellular matrix (ECM) and immune cell composition in their respective microenvironment. GIN was characterized by higher expression level of collagen-related genes, such as COL1 and COL3. Its immune microenvironment was featured by lower proportion of M1 macrophages and notably downregulated complement (C1, C3, C6 and C7) and coagulation cascade. Further analysis of intracellular regulators highlighted reprogrammed metabolism, including retinol metabolism. Downregulated retinoic acid synthesis genes and upregulated catabolic genes indicated depleted retinoic acid in GIN.

**Discussion:**

In conclusion, GIN and ALV exhibited remarkably different extracellular and intracellular characteristics, in particular ECM, immune microenvironment and metabolism, which in turn provides biomechanical environment for epithelial keratinization. This comprehensive study sheds light on mechanisms regulating mucosal keratinization by connective tissues and lays foundation for therapeutic development in regenerative therapies.

## 1 Introduction

**FIGURE 1:**
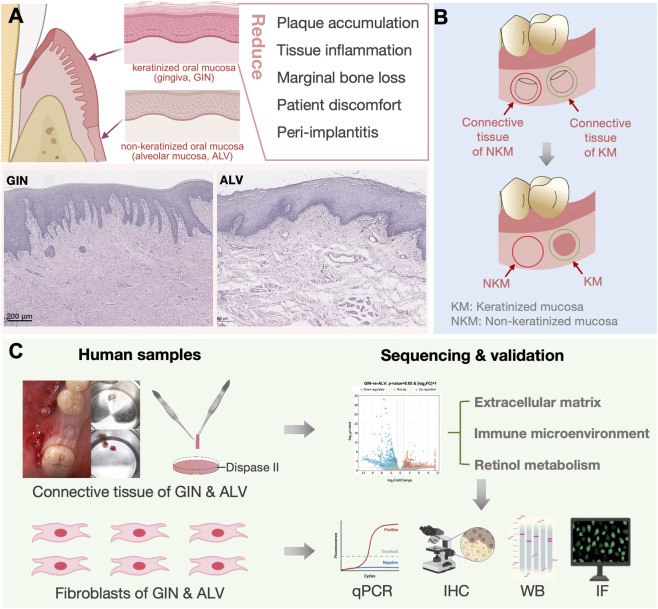
Research background and study design. **(A)** Keratinized and non-keratinized mucosa are anatomically adjacent in the oral cavity yet exhibit significant differences in tissue characteristics. **(B)** Connective tissue intrinsically determines mucosal phenotypes. **(C)** The workflows of this study.

In the oral cavity, keratinized and non-keratinized mucosa are anatomically adjacent but exhibit marked differences in histological characteristics and biological functions (Moutsopoulos and Konkel, 2018; Williams et al., 2021; Larjava et al., 2011; Rojas et al., 2021; Kabakov et al., 2021). Keratinized mucosa plays a crucial role in maintaining the health of periodontal and peri-implant tissue. It has been reported that insufficient keratinized mucosa was associated with increased patient discomfort, plaque accumulation, soft-tissue inflammation, marginal bone loss, and a higher prevalence of peri-implantitis (Ramanauskaite et al., 2022) (Figure 1A). Accordingly, increasing the width of keratinized mucosa has become a major clinical focus in contemporary periodontal and implant therapy.

Free gingival grafting (FGG) remains the gold standard for keratinized mucosa augmentation. However, its application is limited by donor-site morbidity, restricted tissue availability and suboptimal esthetic outcomes. In recent years, various biomaterials have been introduced as alternative strategies, but their capacity to increase keratinized mucosa remains inferior to that of FGG. Owing to the lack of intrinsic biological activity to actively modulate mucosal keratinization, the effectiveness of these materials largely depends on the presence of adjacent pre-existing keratinized mucosa. Consequently, they are unsuitable for cases with extremely limited or completely absent keratinized mucosa. These limitations highlight an urgent clinical need to develop therapeutic agents or bioactive biomaterials capable of actively inducing mucosal keratinization, which in turn requires a comprehensive understanding of the regulatory mechanisms governing oral mucosal keratinization.

Notably, keratinized free gingival graft always retains its phenotype when integrated into a non-keratinized region, indicating that mucosal keratinization is primarily determined by intrinsic genetic factors rather than the local environment(Tavelli et al., 2021). Recently, increasing evidence points to the importance of connective tissue in determining mucosal phenotype(Hsieh et al., 2010). An animal study demonstrates that when connective tissue from keratinized mucosa was transplanted into a non-keratinized region, it retains its keratinized properties, with the newly formed epithelium also exhibits keratinized features (Karring et al., 1975) (Figure 1B). These results emphasize the pivotal role of connective tissue in determining keratinized and non-keratinized oral mucosal phenotypes, which could unlock new, targeted approaches for modulating mucosal phenotypes and advancing regenerative therapies.

Current knowledge regarding the connective tissues underlying keratinized and non-keratinized oral mucosa is largely based on histological observations. In recent years, increasing attention has been directed toward in-depth molecular characterization of these tissues. Several studies have examined gene expression profiles of fibroblasts derived from these tissues. However, considering the pivotal role of connective tissue as a functional unit in regulating oral mucosal keratinization, the intrinsic differences between keratinized and non-keratinized connective tissues themselves remain an important yet insufficiently explored question. To date, there is still a lack of systematic gene expression–based analyses addressing the inherent genetic characteristics of keratinized versus non-keratinized connective tissues.

To address this knowledge gap, the present study aims to investigate the molecular profiles of human keratinized (gingiva, GIN) and non-keratinized (alveolar mucosa, ALV) oral mucosa by analyzing *in situ* connective tissues and isolated fibroblasts (Figure 1C). Bioinformatics analyses identified distinct ECM organization and immune microenvironment features between the two tissue types. Furthermore, intrinsic regulatory network analysis highlighted metabolic reprogramming, particularly alterations in retinol metabolism, as a potential determinant of mucosal phenotype. Collectively, this study established a molecular blueprint keratinized versus non-keratinized connective tissues and provides a foundation for elucidating the mechanisms governing oral mucosal keratinization and phenotype regulation.

## 2 Materials and methods

### 2.1 Sample harvesting

Gingiva and alveolar mucosa samples were obtained from healthy individuals undergoing dental implant surgery or tooth extraction at Hospital of Stomatology, Sun Yat-sen University in 2022. The study was approved by the Medical Ethics Committee of Hospital of Stomatology, Sun Yat-sen University (KQEC-2021–73-02), and all participants provided informed consent. The inclusion and exclusion criteria were as follows:

Inclusion Criteria:

1. Individuals aged 18–40 years;
2. Need for dental implant restoration or tooth extraction;
3. Healthy gingiva with no signs of acute or chronic inflammation;
4. Keratinized mucosa width ≥8 mm at the implant or extraction site;
5. Adequate bone volume at the implant site, without the need for bone augmentation.

Exclusion Criteria:

1. Poor oral hygiene;
2. Uncontrolled oral or systemic diseases;
3. Metabolic disorders such as diabetes;
4. Smoking index >200;
5. Individuals unwilling to participate.

### 2.2 Sample processing

Gingival tissue (GIN) was obtained from the alveolar crest, at least 2 mm from the adjacent teeth, while alveolar mucosa (ALV) was obtained as close to the mucosal junction as possible (Supplementary Figure S8). The samples were treated with 2.4 mg/mL Dispase II solution. The epithelium was discarded, and the connective tissue was immersed in the RNAlater.

### 2.3 RNA extraction and sequencing

RNA was extracted using an RNA extraction kit. RNA purity and quantity were assessed using a NanoDrop 2000 spectrophotometer (Thermo Scientific), with quality standards set at RIN ≥7 and 28S/18S ratio ≥0.7. mRNA was enriched using magnetic beads (Vazyme, N401-2), and cDNA libraries were prepared with the NEBNext Ultra II RNA Library Prep Kit (NEB, E7765). Library concentration and fragment size were evaluated with Qubit and Agilent 2100 systems, respectively. Sequencing was performed on an Illumina Novaseq 6000 platform.

### 2.4 Bioinformatics analysis

Raw sequencing data were processed using fastp software to filter low-quality reads, resulting in Clean reads for analysis. HISAT2 was used for genome alignment. Gene expression levels were calculated using the FPKM method, and read counts for each gene were obtained using HTSeq-count. Differentially expressed genes (DEGs) were identified with DESeq2, applying a p-value threshold of <0.05 and a Foldchange >2 or <0.5.

Volcano plots and heat maps were generated using the Ouyi Biological Cloud Platform (https://cloud.oebiotech.com/task/). Principal Component Analysis (PCA), Gene Ontology (GO) enrichment analysis, KEGG pathway analysis, and Gene Set Enrichment Analysis (GSEA) were conducted. Protein-Protein Interaction (PPI) networks were analyzed using the STRING database, with ClueGO enrichment analysis and Hub gene identification performed using Cytoscape and the MCODE plugin. Immune cell composition was estimated using ImmuCellAI 2.0 based on immune-related gene expression profiles from bulk RNA-seq data. Data visualization was done using the OmicStudio Cloud Platform (https://www.omicstudio.cn/tool), TBtools software and R studio.

### 2.5 RT-qPCR of tissue samples

Tissue samples stored in RNAlater were transferred to RNAse-free EP tubes, homogenized with RNAzol and RNAse-free zirconia grinding beads, and vortexed. The mixture was centrifuged at 12,000 g for 15 min. The supernatant was collected, and RNA was precipitated with 75% ethanol. The pellet was washed twice with 75% ethanol, air-dried for 5 min, and resuspended in DEPC water. RNA concentration was measured with a micro-spectrophotometer and stored at −80 °C. RNA was reverse transcribed using the Hifair® III first Strand cDNA Synthesis SuperMix Kit. RNA, RNase-Free H2O, and gDNA digester Mix were incubated at 42 °C for 2 min to remove genomic DNA. Hifair® III SuperMix plus was added, and the mixture was incubated at 25 °C for 5 min, 55 °C for 15 min, and 85 °C for 5 min. The cDNA was stored at −20 °C. Primers were designed based on cDNA sequences from NCBI and validated in the BLAST database. GAPDH was used as a reference gene. Gene expression was assessed using SYBR real-time quantitative PCR premix on a 384-well plate. The two-step reaction protocol was employed, and data were analyzed using the 2^−ΔΔCT^ method. Primer sequences are provided in Supplementary Table S1.

### 2.6 Histological staining

Tissue samples were fixed in 4% paraformaldehyde for 24 h, followed by dehydration, embedding, and sectioning. The slices were deparaffinized in xylene and rehydrated through a graded ethanol series. Hematoxylin and eosin (H&E) staining, Masson’s trichrome staining, and Elastic Van Gieson (EVG) staining were performed according to standard protocols. After staining, the sections were dehydrated, mounted, and observed under a light microscope (Carl Zeiss, Germany).

### 2.7 Immunohistochemistry staining

The slices were treated with heat-induced antigen retrieval and incubated in 3%H2O2 for 15 min. After blocking with bovine serum albumin for 1 h, the slices were incubated with COL1 (Abcam, Cambridge, UK; Cat# ab138492; 1:750), COL3 (Abcam, Cambridge, UK; Cat# ab184993; 1:100), ADH (Abcam, Cambridge, UK; Cat# ab108203; 1:500), CYP26B1 (Affinity, Cincinnati, OH, USA; Cat# DF12194; 1:200), AOX1 (Affinity, Cincinnati, OH, USA; Cat# DF3756; 1:200), and C3 (Affinity, Cincinnati, OH, USA; Cat# DF13224; 1:200) overnight at 4 °C, incubated with a goat anti-rabbit IgG secondary antibody (Gene Tech, Shanghai, China; Cat# GK600710) and counterstained with hematoxylin for 1 min. The slices were imaged using an Axio Imager M2 microscope (Carl Zeiss). Semi-quantitative analysis was performed using Image-Pro Plus software. For each slice, five random fields were selected, positive cells were segmented based on OD values, and density was calculated by IOD/area.

### 2.8 Multiplex immunofluorescence

The slices were deparaffinized, rehydrated, and subjected to heat-induced antigen retrieval in citrate buffer (pH 6.0). After autofluorescence quenching and blocking with 3% BSA, the sections were incubated overnight at 4 °C with primary antibodies against ADH (1:200), CYP26B1 (1:100), AOX1 (1:100), and C3 (1:100), followed by incubation with Alexa Fluor® 488-conjugated secondary antibodies (Abcam, Cambridge, UK; Cat# ab150077; 1:400) for 50 min at room temperature in the dark. The sections were then incubated with antibodies against CD68 (Abcam, Cambridge, UK; Cat# ab213363; 1:1000) or CD31 (Abcam, Cambridge, UK; Cat# ab182981; 1:1000), followed by Alexa Fluor® 555-conjugated secondary antibodies (Abcam, Cambridge, UK; Cat# ab150078; 1:400) for 50 min at room temperature in the dark. Nuclei were counterstained with DAPI, and the sections were observed under a fluorescence microscope (Nikon, Japan).

### 2.9 Primary fibroblasts culturing

Gingiva and alveolar mucosa were collected and processed within 30 min. The tissues were treated with 2.4 mg/mL Dispase II solution, incubated at 37 °C for 2.5 h, and then separated into epithelial and connective tissue. Connective tissue was digested with 0.25% trypsin and 0.5% collagenase I, filtered, and cultured in DMEM containing 15% fetal bovine serum and 3% penicillin/streptomycin. Cultured fibroblasts derived from gingiva and alveolar mucosa were designated as human gingival fibroblasts (HGF) and human alveolar mucosal fibroblasts (HAMF), respectively.

### 2.10 RT-qPCR of cultured fibroblasts

RNA from HGF and HAMF was extracted using an RNA rapid extraction kit according to the manufacturer’s instructions. Reverse transcription and RT-qPCR were performed as described in Section 2.5.

### 2.11 Western blot

HGF and HAMF were seeded into 6-well plates and cultured until cells reached confluence. Cells were lysed using RIPA lysis buffer containing 1% protease inhibitor and incubated on ice for 30 min. The lysates were centrifuged at 15,000 × g for 15 min at 4 °C, and the supernatants were collected. Protein concentrations were measured using the BCA Protein Assay Kit and diluted to 1 μg/μL. After denaturation for 10 min, the samples were subjected to SDS-PAGE electrophoresis using 10% Bis-Tris precast gels (145 V, 45 min). Proteins were transferred to PVDF membranes (400 mA, 40 min) and blocked with 5% skim milk for 1 h at room temperature. The membranes were incubated overnight at 4 °C with primary antibodies (dilutions: Actin 1:1000, COL1 1:5000, COL3 1:2000, ADH 1:5000, AOX1 1:1000, CYP26B1 1:1000). After washing with TBST, membranes were incubated with secondary antibodies at room temperature for 45 min. Signals were developed using an ECL detection reagent and visualized with a chemiluminescence imaging system.

### 2.12 Immunofluorescence

HGF and HAMF were seeded onto glass coverslips and cultured to appropriate density. Cells were fixed with 4% paraformaldehyde for 15 min, washed with PBS, and permeabilized with 0.3% Triton X-100 for 15 min, followed by PBS washes. Cells were blocked with 1% BSA for 1 h at room temperature and incubated overnight at 4 °C with primary antibody (dilutions: COL1 1:1000, COL3 1:200, ADH 1:1000, AOX1 1:500, CYP26B1 1:500). The next day, samples were rewarmed at room temperature for 30 min, washed with PBS, and incubated with a fluorescent secondary antibody for 1 h in the dark. Actin green fluorescent probe (30 min) and DAPI (5 min) were used for staining, with all steps performed in the dark. After washing with PBS and ultrapure water, coverslips were mounted with an anti-fade reagent and stored at 4 °C in the dark. Samples were observed and imaged using a confocal laser scanning microscope.

## 3 Results

### 3.1 Transcriptomic analysis revealed extensive molecular differences between the GIN and ALV

To dissect the profiles of keratinized (GIN) and non-keratinized (ALV) connective tissue, genome-wide transcriptomic sequencing was performed. The boxplot of FPKM values reveals a generally consistent distribution of gene expression levels across samples, with the ALV group demonstrating greater consistency (Supplementary Figure S1A). Principal Component Analysis (PCA) illustrates distinct spatial separation between GIN and ALV samples, indicating significant differences between the two groups. Specifically, ALV samples cluster more closely together, whereas GIN samples exhibit greater dispersion, reflecting increased heterogeneity within the GIN group (Supplementary Figure S1B). Differential expression analysis identified 1,309 genes with significant expression changes (p-value<0.05 & |log2FC|>1), including 480 upregulated genes and 829 downregulated genes in GIN compared to ALV. Heatmaps visually represent the extensive differences in gene expression profiles between GIN and ALV, while volcano plots further illustrate the distribution of differentially expressed genes between the two groups (Supplementary Figures S1C,D).

### 3.2 The extracellular matrix (ECM) composition emerged as the primary distinction between the molecular profiles of GIN and ALV

**FIGURE 2:**
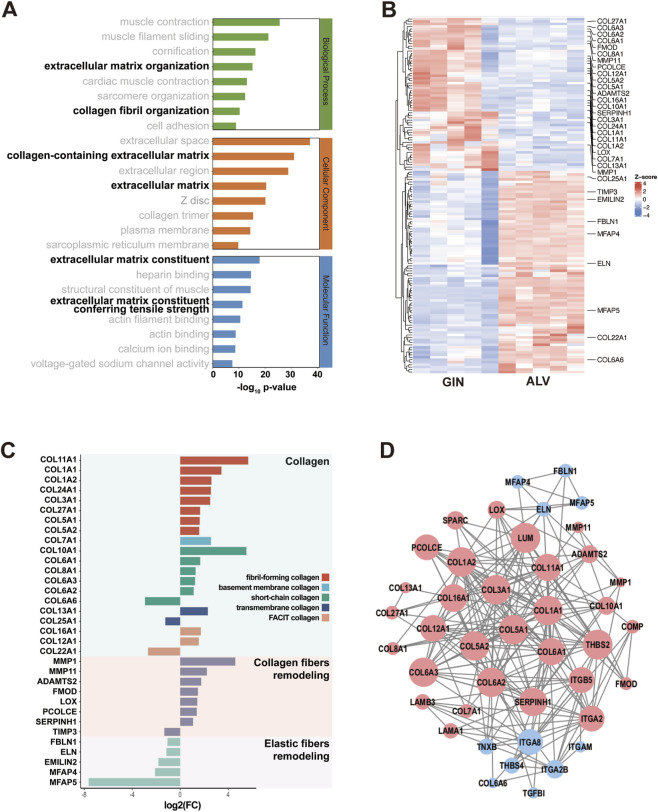
Differential expression analysis of extracellular matrix-related genes between GIN and ALV. **(A)** GO enrichment analysis based on all differentially expressed genes showing the top eight terms with the lowest p-values. **(B)** Differential expression of extracellular matrix-related genes. **(C)** Differential expression and classification of key extracellular matrix-related genes. The bar chart shows the fold changes in gene expression in GIN relative to ALV. **(D)** PPI analysis of extracellular matrix-related genes, with the most relevant cluster visualized by MCODE. Blue circles indicate genes downregulated in GIN, and red circles indicate upregulated in GIN.

To dissect the major molecular difference between GIN and ALV, functional enrichment analysis was performed based on genome-wide transcriptomic results. GO enrichment analysis of differentially expressed genes initially revealed a predominant prevalence of terms associated with the extracellular matrix across Biological Process, Cellular Component, and Molecular Function categories, highlighting 726 enriched terms. Notably, most of the enriched terms were related to the extracellular matrix (Figure 2A). The heatmap of differentially expressed extracellular matrix–related genes revealed substantial upregulation in both the GIN and ALV; however, these genes segregate into distinct functional modules. Specifically, genes upregulated in GIN are predominantly enriched in collagen biosynthesis–related processes, whereas those upregulated in ALV are primarily associated with elastin organization and regulation (Figures 2B,C).

Protein-protein interaction (PPI) analysis followed by MCODE clustering identified core genes upregulated in GIN primarily related to collagen, including fibril-forming collagens (e.g., COL1A1, COL1A2, COL3A1, COL5A1), basement membrane collagen (COL7A1), short-chain collagens (e.g., COL10A1, COL6A1, COL8A1), and genes associated with collagen fiber synthesis and remodeling (e.g., MMP1, MMP11, ADAMTS2, FMOD, LOX, PCOLCE, and SERPINH1). Conversely, genes related to elastin fibers and their synthesis and remodeling, including ELN1, FBLN1, MFAP4, and MFAP5, were upregulated in ALV (Figure 2D).

**FIGURE 3:**
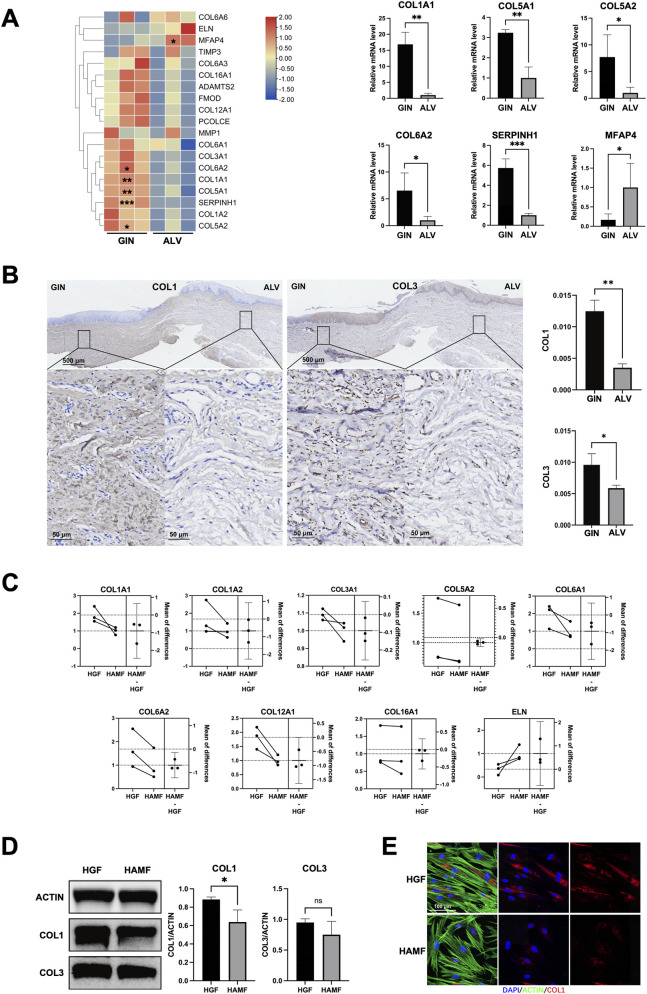
**(A)** RNA expression level of key extracellular matrix-related genes in GIN and ALV determined by RT-qPCR. **(B)** Immunohistochemical staining of COL1 and COL3 in GIN and ALV. Representative images show the localization and intensity of staining. **(C)** Expression profiles of collagen and elastin-related genes in fibroblasts derived from GIN (HFG) and ALV (HAMF), assessed via quantitative RT-qPCR. Data are presented as mean ± SD. **(D)** Representative bands and semi-quantitative analysis of Western blot for COL1 and COL3 in fibroblasts from GIN and ALV. **(E)** Immunofluorescence staining for COL1 in fibroblasts originating from GIN and ALV, with representative images displaying the cellular distribution of COL1. Data represent means ± SD from three independent experiments. Significant differences to the control are indicated as ***p < 0.001, **p < 0.01, *p < 0.05.

Heatmap of RT-qPCR validation corroborated the transcriptome sequencing results (Figure 3A). Specifically, GIN exhibited significantly higher expression of COL1A1, COL5A1, COL5A2, COL6A2, and the collagen synthesis-related gene SERPINH1 compared to ALV. Conversely, MFAP4, involved in elastin fiber assembly, was significantly less expressed in GIN (Figure 3A). Immunohistochemistry (IHC) staining revealed widespread expression of Type I collagen (COL1) in the extracellular matrix of GIN, with semi-quantitative analysis indicating significantly higher COL1 expression in GIN compared to ALV (p = 0.009). Type III collagen (COL3) was expressed sparsely in both GIN and ALV but at significantly higher levels in GIN (p = 0.049) (Figure 3B; Supplementary Figure S2).

Verification at cellular levels showed that collagen-related genes—COL1A1, COL1A2, COL3A1, COL5A2, COL6A1, COL6A2, COL12A1, and COL16A1—were more highly expressed in gingival fibroblasts (HGF) compared to alveolar mucosal fibroblasts (HAMF), whereas elastin (ELN) expression was lower in HGF (Figure 3C). Western blot analysis revealed significantly higher COL1 expression in HGF compared to ALV (p = 0.033), while COL3 expression did not differ significantly (Figure 3D; Supplementary Figure S3). Immunofluorescence staining further demonstrated that COL1 expression is notably higher in HGF compared to HAMF (Figure 3E; Supplementary Figure S4). Taken together, our results showed markedly different ECM components in these connective tissues indicating matrix stiffness and mechanical signaling might be important for keratinization.

### 3.3 Distinct immune microenvironment features in GIN and ALV

**FIGURE 4:**
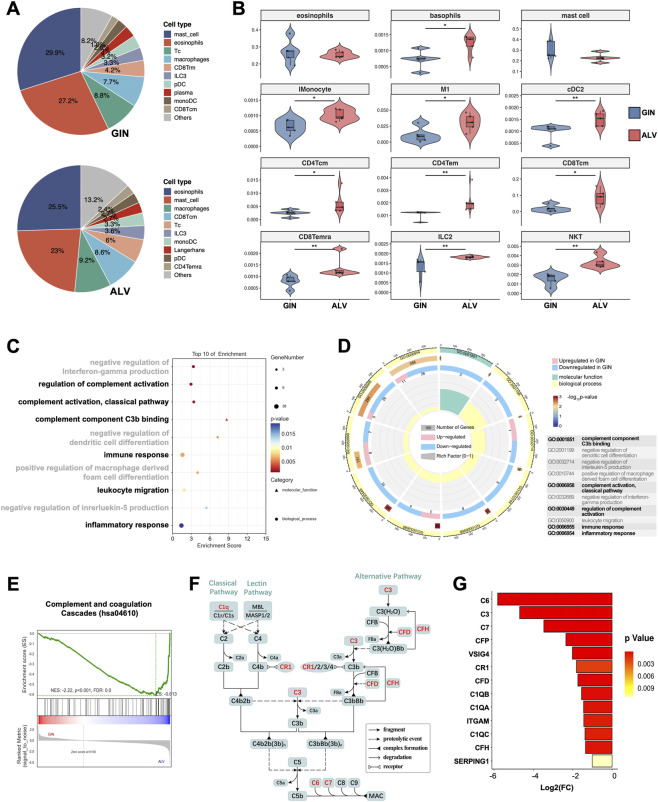
Differential expression analysis of immune-related genes between GIN and ALV. **(A)** Immune cell composition in GIN and ALV. **(B)** Differences of specific immune cell subtypes between GIN and ALV. **(C)** GO enrichment analysis showing the top 10 immune-related terms with the lowest p-values based on all differentially expressed genes. **(D)** GO enrichment circle plot for immune-related terms (First circle: Categories and terms; Second circle: Number of genes and p-values; Third circle: Ratio of upregulated to downregulated genes; Fourth circle: RichFactor values, reflecting the degree of enrichment). **(E)** GSEA plot for the complement and coagulation cascade pathways. **(F)** Flowchart illustrating the complement activation pathways, with significantly differentially expressed genes highlighted in red. **(G)** The bar chart shows fold changes (GIN relative to ALV) and p-values for complement-related genes. Bar chart displaying fold changes and p-values for differentially expressed complement-related genes. Significant differences between GIN and ALV are indicated: ***p < 0.001, **p < 0.01, *p < 0.05.

Distinct ECM between keratinized and non-keratinized mucosal connective tissues prompts us to ask if other microenvironment features are also different. Computational inference of immune cell composition in GIN and ALV suggested that eosinophils and mast cells were among the predominant immune cell populations in both tissues, followed by macrophages (Figure 4A). Inferred compositions of specific myeloid and lymphoid immune cell populations appeared to differ between GIN and ALV. Notably, inferred M1 macrophage abundance appeared to be higher in ALV (Figure 4B). Differential expression heatmap analysis demonstrated that immune-related genes were predominantly downregulated in GIN compared with ALV (Supplementary Figure S5A). ClueGO functional enrichment analysis further indicated that the key pathways and genes are mainly involved in complement activation and T-lymphocyte chemotaxis (Supplementary Figure S5B).

Consistently, GO analysis identified significant enrichment of complement-related terms (Figure 4C). The GO enrichment circle plot showed uniform downregulation of complement pathway genes in GIN (Figure 4D). In addition, GSEA revealed downregulated gene sets in GIN were associated with the complement and coagulation cascade pathways (Figure 4E). Further analysis confirmed substantial downregulation of complement activation–associated genes in GIN. Specifically, the core components C3, C6 and C7 expression were 26-, 56.37- and 11.09-fold higher in ALV, respectively (Figures 4F,G).

**FIGURE 5:**
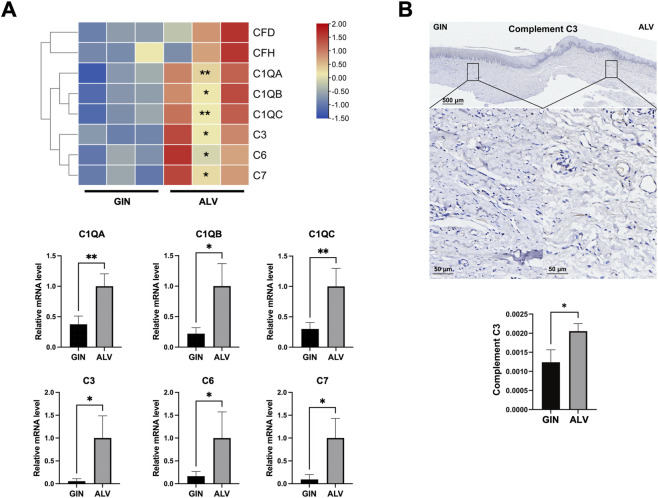
**(A)** Heatmap and bar chart showing RT-qPCR results for key complement system genes, highlighting expression differences between GIN and ALV. **(B)** Immunohistochemical staining of C3 in GIN and ALV. Data represent means ± SD from three independent experiments for RT-qPCR and Western blot, and from five samples per tissue type for immunofluorescence. Significant differences between GIN and ALV are indicated: ***p < 0.001, **p < 0.01, *p < 0.05.

RT-qPCR validation of key genes in the complement system confirmed that ex-pression trends align with transcriptome sequencing results. Specifically, complement components C1QA (*p* = 0.006), C1QB (*p* = 0.012), C1QC (*p* = 0.009), C3 (*p* = 0.038), C6 (*p* = 0.034), and C7 (*p* = 0.012) were all significantly upregulated in ALV (Figure 5A). IHC staining results suggested that the expression level of complement component C3 was significantly higher in ALV compared to GIN (p = 0.046) (Figure 5B; Supplementary Figure S2). Co-staining of C3 and CD31 further demonstrated that C3 was predominantly expressed in superficial vascular endothelial cells, while its expression in macrophages was relatively low (Supplementary Figure S6). These findings suggested that ECM accompanied with immune cells composition might be reshaped in various kinds of connective tissues.

### 3.4 The retinol metabolism pathway, which is closely associated with keratinization, exhibited significant differences between GIN and ALV

**FIGURE 6:**
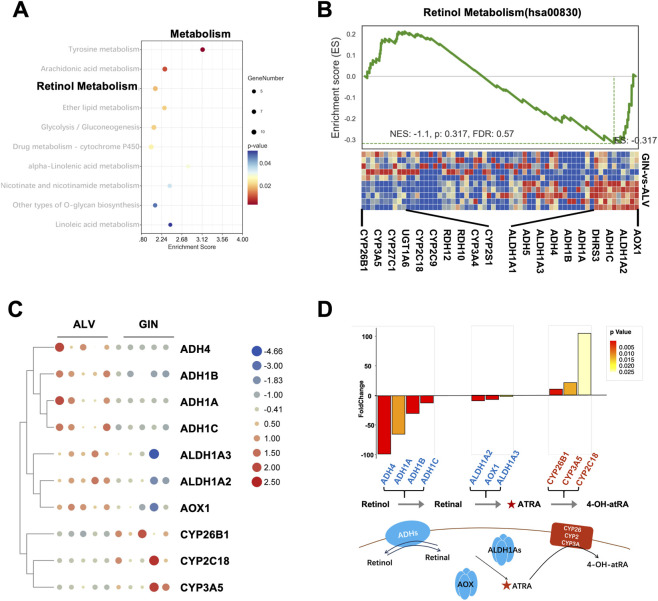
Differential expression analysis of retinol metabolism-related genes between GIN and ALV. **(A)** Top 10 pathways from KEGG enrichment analysis ranked by p-value based on all differentially expressed genes. **(B)** GSEA plot for retinol metabolism pathways, with heatmap displaying expression of pathway-specific genes. Red indicates upregulated genes, whereas blue indicates downregulated genes. **(C)** Differential expression of key genes across GIN and ALV samples. Red indicates upregulated genes, whereas blue indicates downregulated genes. Dot size represents the relative expression level of each gene in the samples. **(D)** The schematic illustrates gene functions within the pathway, with genes upregulated in GIN shown in red and downregulated genes in blue. The bar chart presents the fold changes and p-values of key genes in the vitamin A metabolism pathway. ATRA: all-trans retinoic acid.

We further investigate underlying mechanisms that modulate the formation of different microenvironment. KEGG analysis revealed significant enrich of the retinol metabolism pathway, which has been shown to play a critical role in epithelial keratinization (Figure 6A). Its key metabolite, retinoic acid, may regulate keratinization by directly secreting into epithelial layer or modulating the secretory phenotype of connective tissue to reshape the microenvironment. GSEA results indicated that upstream genes of this pathway were significantly upregulated in GIN, whereas downstream genes were downregulated (Figure 6B). Further examination showed that genes involved in retinoic acid synthesis, including alcohol dehydrogenases (ADH1A, ADH1B, ADH1C, ADH4), aldehyde dehydrogenases (ALDH1A2, ALDH1A3), and aldehyde oxidase (AOX1), exhibited lower expression in GIN compared to ALV. Conversely, genes related to retinoic acid metabolism, such as cytochrome P450 enzymes (CYP26B1, CYP2C18, CYP3A5), were upregulated in GIN (Figures 6C,D).

**FIGURE 7:**
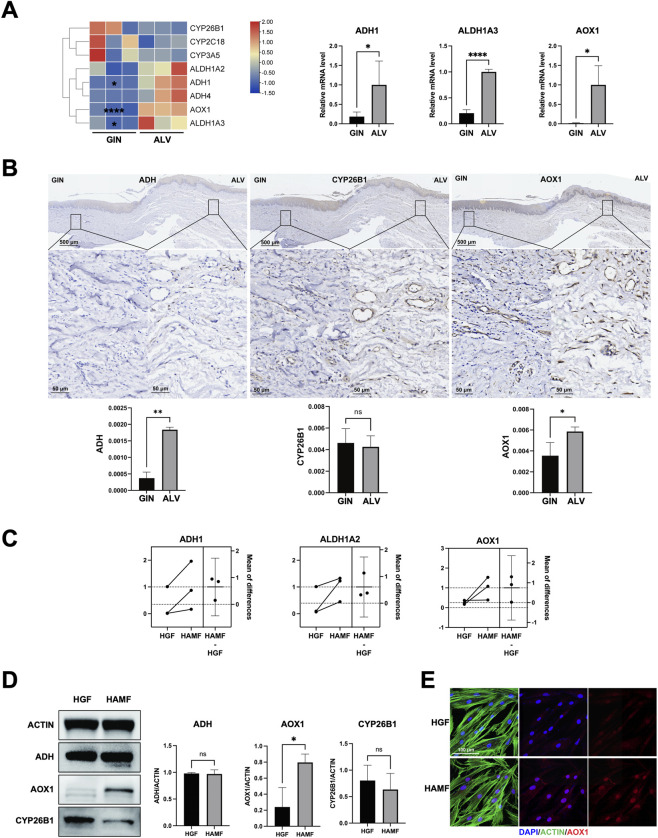
**(A)** Heatmap and bar chart showing RT-qPCR validated differential expression of key vitamin A metabolism genes in GIN and ALV. **(B)** Immunohistochemical staining of ADH, AOX1, and CYP26B1 in GIN and ALV. **(C)** RT-qPCR profiles of retinol metabolism-related genes in fibroblasts from GIN and ALV. **(D)** Representative bands and semi-quantitative analysis of Western blot for ADH, AOX1, and CYP26B1 in fibroblasts from GIN and ALV. **(E)** Immunofluorescence staining of AOX1 in fibroblasts from GIN and ALV. Data represent means ± SD from three independent experiments. Significant differences between GIN and ALV are indicated: ***p < 0.001, **p < 0.01, *p < 0.05.

RT-qPCR validation confirmed that the expression trends were consistent with transcriptome sequencing results. Specifically, ADH1 (p = 0.037), ALDH1A3 (p = 0.043), and AOX1 (p < 0.001) were significantly upregulated in GIN (Figure 7A). Immunohistochemistry (IHC) staining showed that alcohol dehydrogenase (ADH) was sparsely expressed in ALV, predominantly in fibroblasts, with minimal ADH staining in GIN. Semi-quantitative analysis further revealed significantly higher ADH expression in ALV compared to GIN (p = 0.004). Similarly, aldehyde oxidase 1 (AOX1) was observed in endothelial cells and fibroblasts in both GIN and ALV, with a higher number of AOX1-positive cells in ALV. Semi-quantitative analysis confirmed that AOX1 expression was significantly higher in ALV than in GIN (p = 0.025). Cytochrome P450 26B1 (CYP26B1) was diffusely expressed in both GIN and ALV, with no significant difference in expression levels between the two groups (Figure 7B; Supplementary Figure S2).

Further RT-qPCR validation in fibroblasts (HGF and HAMF) showed that ADH1, ALDH1A2, and AOX1 were more highly expressed in HGF compared to HAMF (Figure 7C). Western blot analysis indicated a significantly lower expression of AOX1 in HGF compared to ALV (p = 0.014), while the expression levels of ADH and CYP26B1 did not exhibit statistically significant differences (Figure 7D; Supplementary Figure S3). Consistently, immunofluorescence staining revealed markedly higher AOX1 expression in HAMF relative to HGF, with no significant differences observed for ADH and CYP26B1 expression (Figure 7E; Supplementary Figure S4). Collectively, our findings indicate that metabolic pathways are reprogrammed in keratinized connective tissues, which may result in the attenuation of retinoic acid secretion into its microenvironment.

## 4 Discussion

Connective tissue has long been recognized as a decisive determinant of oral mucosal phenotype, but its intrinsic molecular characteristics under physiological conditions have remained insufficiently elucidated(Karring et al., 1975). A comprehensive understanding of the differences between keratinized and non-keratinized mucosa is therefore imperative for advancing the exploration of mucosal phenotypes regulatory mechanisms. Here, we addressed this knowledge gap through multifaceted analysis on extracellular and intracellular features of connective tissues from human gingiva and alveolar mucosa. Our findings reveal three interrelated layers of distinction: extracellular matrix organization, immune microenvironment composition, and metabolic reprogramming—particularly retinol metabolism. Together, these features may represent a molecular blueprint potentially involved in the regulation of epithelial keratinization.

Among all differentially expressed pathways, extracellular matrix (ECM) organization emerged as the most prominent distinction between GIN and ALV. This finding is consistent with histological observations (Supplementary Figure S7) and mechanistic studies suggesting that specific ECM components influence epithelial differentiation (Nguyen et al., 2019; Komori et al., 2018; Hsieh et al., 2010; Mu et al., 2024). Further analysis of differentially expressed genes revealed that type I and type III collagen, the major structural proteins of connective tissue, exhibited gene expression levels more than five times higher in keratinized mucosa compared to non-keratinized mucosa. The significant upregulation suggests a prominent role of these collagens in maintaining the structural integrity and function of keratinized mucosa. Furthermore, genes expression of collagens that play important roles in collagen fiber assembly and functional regulation of cells were highly expressed in gingiva (Wenstrup et al., 2004; Fitzgerald et al., 2013; Lettmann et al., 2014; Theocharidis et al., 2016; Izu and Birk, 2023). Genes expression of enzymes that positively regulate collagen remodeling were also upregulated in gingiva (Ito and Nagata, 2017). Conversely, TIMP3, a tissue inhibitor of metalloproteinases, was downregulated in gingiva. These results suggest that collagen fibers in gingiva may have a more active synthesis and metabolic process. Given that matrix stiffness and mechanotransduction are known regulators of epithelial cell fate, it is plausible that a collagen-dominant, mechanically rigid microenvironment in GIN provides a biomechanical niche conducive to keratinization Nevertheless, the association between the structure, metabolic process, or other specific components of the connective tissue extracellular matrix and epithelial keratinization has not been delineated. This study provides potential directions and a theoretical basis for further mechanistic exploration.

Beyond structural differences, we observed distinct immune microenvironment profiles. Although eosinophils and mast cells predominated in both tissues, ALV exhibited significantly higher proportions of pro-inflammatory M1 macrophages. More strikingly, complement cascade genes—including C1q subunits, C3, C6, and C7—were markedly upregulated in ALV at both transcriptomic and protein levels. The complement system is a central mediator of innate immunity and inflammatory amplification. Given that ALV lacks a keratinized epithelial barrier, it is likely exposed to greater microbial and mechanical challenges, necessitating heightened immune surveillance(Moutsopoulos and Konkel, 2018). However, excessive complement activation has been implicated in tissue destruction and periodontal inflammation(Moutsopoulos and Konkel, 2018; Damgaard et al., 2015; Huang et al., 2023). Therefore, the relatively quiescent complement profile observed in GIN may be associated with its superior resistance to inflammation and its clinically documented protective role against peri-implant disease and marginal bone loss(Ramanauskaite et al., 2022; Ravidà et al., 2022). These findings suggest a potential association between the clinical benefit of keratinized mucosa and differences in the innate immune environment of the underlying connective tissue, in addition to mechanical characteristics.

Metabolic pathway analysis further revealed significant differences in retinol metabolism, a pathway intimately associated with epithelial differentiation. Retinoic acid (RA), the active derivative of vitamin A, is well established as an inhibitor of keratinization(Szymański et al., 2020; Törmä, 2011; Virtanen et al., 2000; Li et al., 2019). *In vitro* studies have demonstrated that fetal bovine serum suppresses keratinization of mouse epithelial cells, and the effect could be reversed by retinoic acid receptor inhibitor(Ozaki et al., 2019). Additionally, Miyazono et al. confirmed that RA inhibits keratinization of epithelial cells across multiple species, including mice, pigs, and humans(Miyazono et al., 2020). In retinol metabolic pathway, retinol is oxidized to retinal by alcohol dehydrogenase and subsequently converted to retinoic acid by aldehyde dehydrogenase and aldehyde oxidase(Duester, 2001; Zhong et al., 2021; Molotkov et al., 2002). Retinoic acid is then catabolized by cytochrome P450 enzymes, primarily CYP26 (Veit et al., 2021; Gudas, 2022). Our data indicated downregulation of genes involved in RA synthesis and upregulation of RA-catabolizing enzymes in GIN. It could be speculated that reduced RA signaling in GIN may remove inhibitory constraints on keratinization, while elevated RA metabolic activity in ALV may actively maintain a non-keratinized phenotype. Thus, retinol metabolism may represent a potential biochemical axis involved in connective tissue–associated regulation of epithelial fate. Interestingly, fibroblast-only validation revealed partial discrepancies compared with whole-tissue data, suggesting that RA metabolism may be influenced by endothelial cells, immune cells, or epithelial–stromal crosstalk rather than being solely fibroblast-autonomous. This observation underscores the importance of studying connective tissue as a multicellular functional unit rather than isolated stromal cells.

**FIGURE 8:**
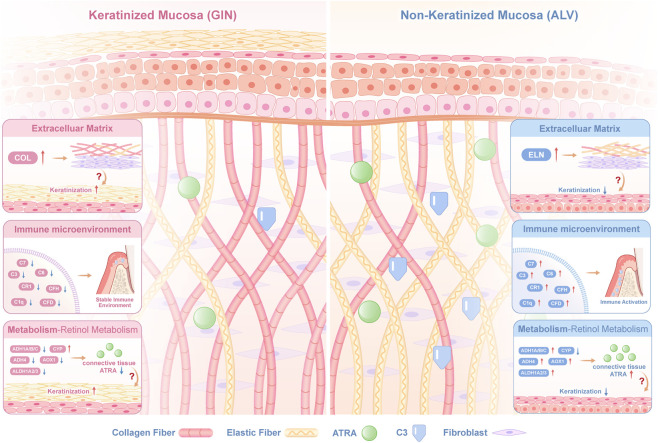
Schematic diagram illustrating distinct connective tissue molecular blueprints in human keratinized and non-keratinized oral mucosa.

By integrating structural, immune, and metabolic findings, we propose a conceptual framework in which connective tissue may influence mucosal phenotype through three coordinated mechanisms: (1) Biomechanical regulation via collagen-dominant ECM composition and increased matrix stiffness in GIN. (2) Immune modulation through restrained complement activation and reduced inflammatory tone. (3) Metabolic signaling via differential retinoic acid synthesis and catabolism influencing epithelial differentiation (Figure 8). Rather than acting independently, these mechanisms may interact, and keratinization should be viewed as the emergent outcome of a coordinated stromal regulatory network.

To our knowledge, this is the first study to perform transcriptomic sequencing of gingival and alveolar mucosal connective tissues in healthy individuals, systematically characterizing their gene expression differences under physiological conditions. The findings provide new directions and evaluation metrics for studying mucosal phenotype modulation and regeneration. Future strategies for mucosal phenotype regulation include altering extracellular matrix composition with defined mechanical properties, modulating immune microenvironment to recreate a low-inflammatory stromal niche and targeting retinoic acid signaling to direct epithelial differentiation. Such approaches may enable bioactive modulation of mucosal phenotype rather than passive tissue replacement, and thus achieving long-term stability and functional maintenance of keratinized tissues.

Despite the valuable insights gained, this study has some limitations. The sample size was limited, and transcriptomic profiling was performed on bulk tissue, precluding precise cell-type resolution. Future studies employing single-cell RNA sequencing or spatial transcriptomics would allow identification of the specific stromal or immune subpopulations driving phenotype determination. Functional experiments manipulating collagen composition, complement signaling, or RA metabolism will also be necessary to establish causality.

## 5 Conclusion

GIN and ALV exhibited remarkably different extracellular and intracellular characteristics, in particular ECM, immune microenvironment and metabolism, which may contribute to distinct biomechanical environments associated with epithelial keratinization. To our knowledge, this study is the first to systematically map the molecular profiles of connective tissue in human keratinized and non-keratinized oral mucosa, providing foundational insights that may guide future research on mucosal phenotype modulation and regenerative therapies.

## Data Availability

The original contributions presented in the study are included in the article/Supplementary Material, further inquiries can be directed to the corresponding authors.
