# Supplementary material for: Connective tissue profiling in keratinized and non-keratinized oral mucosa reveals distinct extracellular and intracellular features
Source: Front Cell Dev Biol. 2026 Jun 12;14:1815527. doi: 10.3389/fcell.2026.1815527 (PMC13304650; doi:10.3389/fcell.2026.1815527)
Supplement: Supplementary file 1 [file DataSheet1.pdf]

Supplementary Material

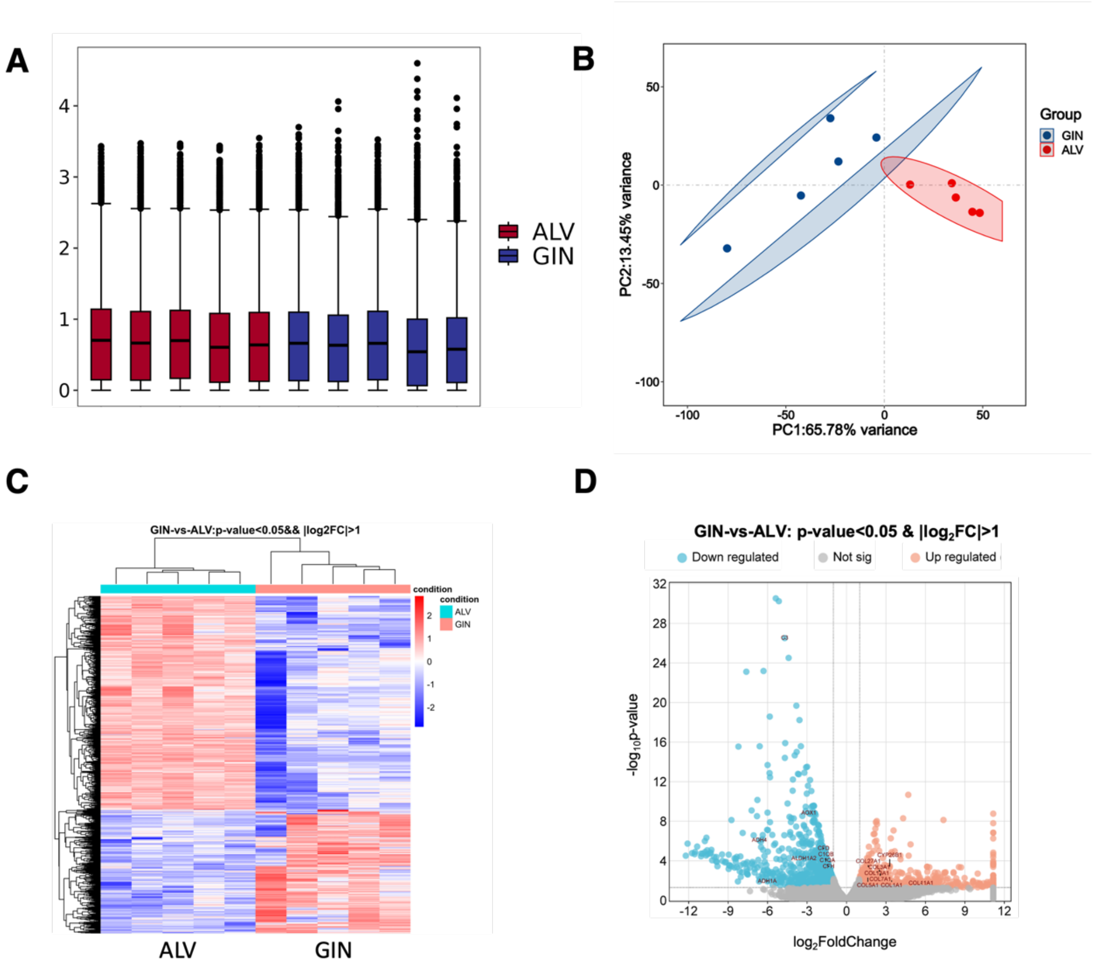

Supplementary Figure 1. Gene expression profiles between gingival (GIN) and alveolar (ALV) mucosa. (A) Boxplot shows gene expression distributions in GIN and ALV samples, with higher median expression in GIN. (B) Principal Component Analysis (PCA) plot visualizing gene expression clustering in GIN versus ALV samples. (C) Heatmap displays gene expression differences between GIN and ALV. (D) Volcano plot showing differentially expressed genes between GIN and ALV.

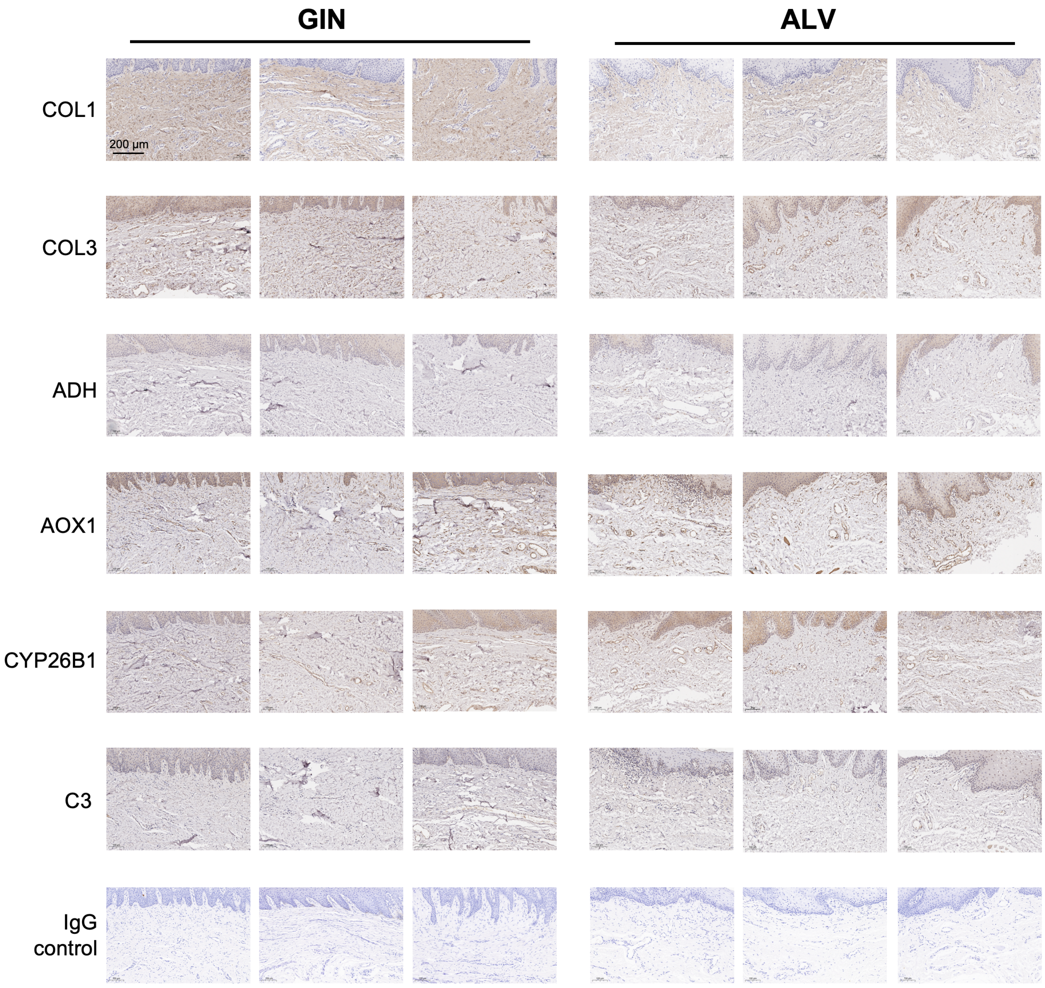

Supplementary Fig 2. Representative images of immunohistochemistry (IHC) staining for GIN and ALV. (COL1: type I collagen. COL3: type III collagen. ADH: alcohol dehydrogenase. AOX1: aldehyde oxidase 1. CYP26B1: cytochrome P450 family 26 subfamily B member 1. C3: complement component 3.)

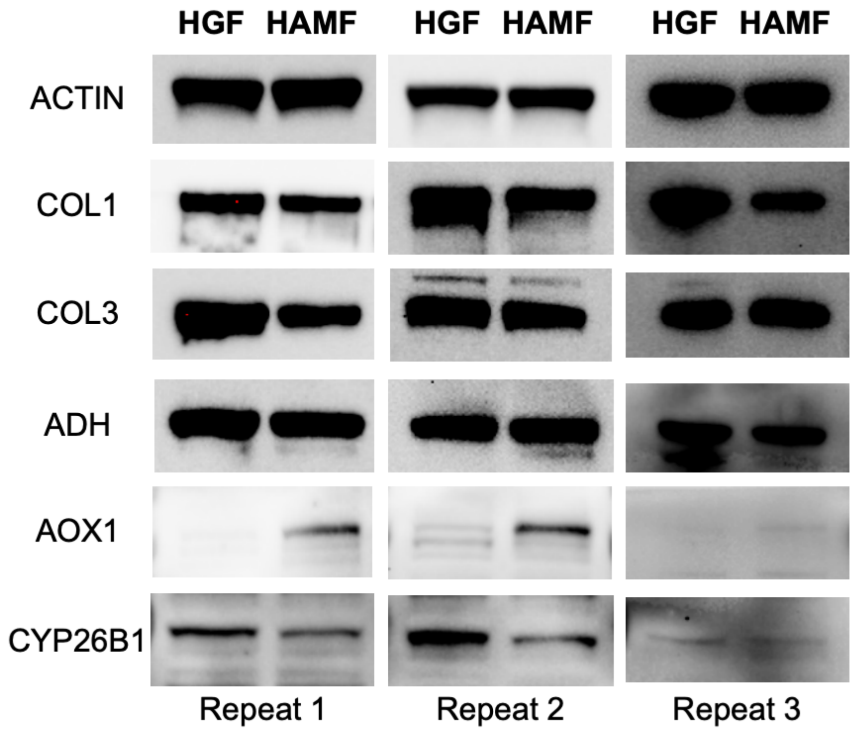

Supplementary Figure 3. Western blot of COL1, COL3, ADH, AOX1 and CYP26B1 in HGF and HAMF. (COL1: type I collagen. COL3: type III collagen. ADH: alcohol dehydrogenase. AOX1: aldehyde oxidase 1. CYP26B1: cytochrome P450 family 26 subfamily B member 1.)

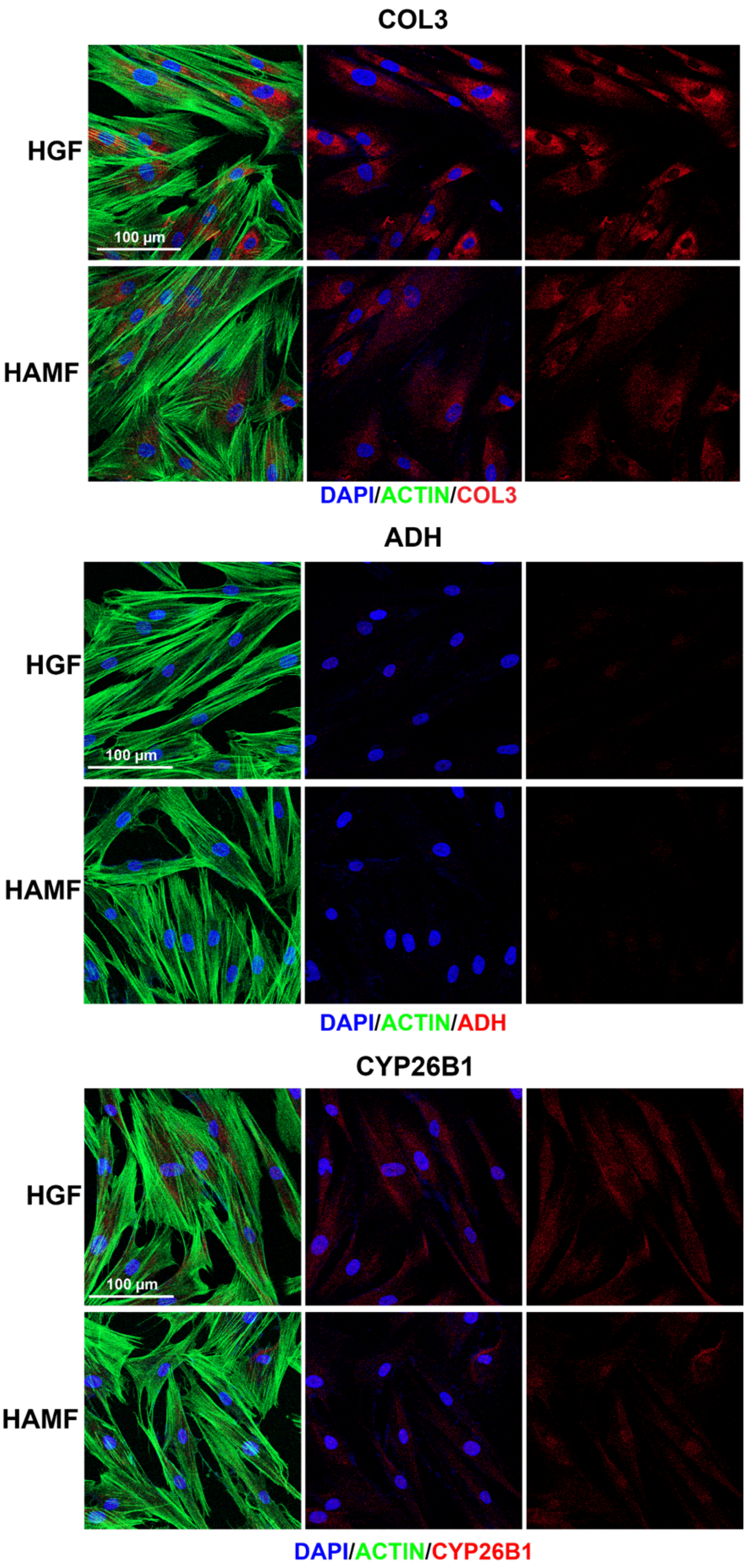

Supplementary Figure 4. Immunofluorescence staining of COL3, ADH and CYP26B1 in HGF and HAMF. (COL3: type III collagen. ADH: alcohol dehydrogenase. CYP26B1: cytochrome P450 family 26 subfamily B member 1.)

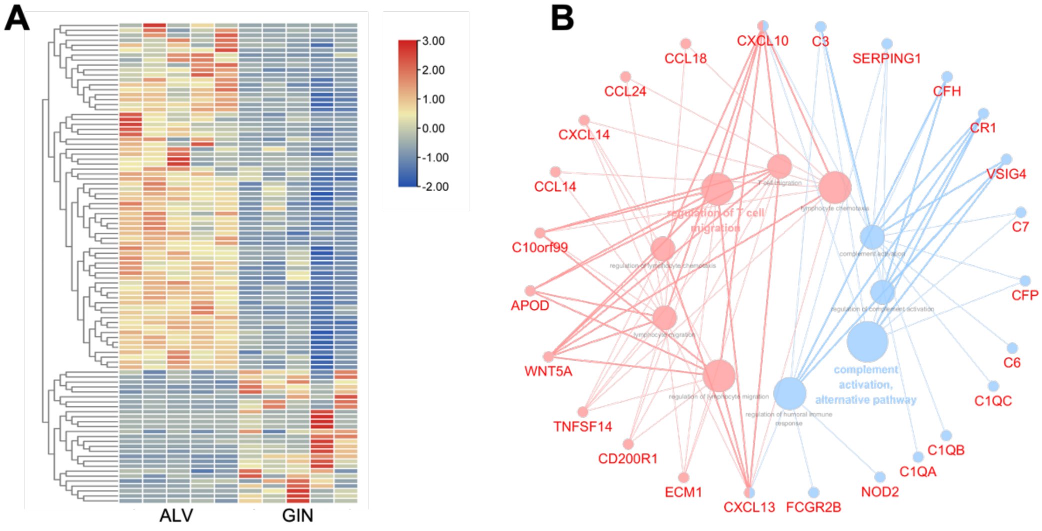

Supplementary Figure 5. (A) Heatmap of immune-related gene expression differences between GIN and ALV. (B) ClueGO functional enrichment analysis indicating that complement activation- and T-lymphocyte chemotaxis-related pathways and associated genes in GIN and ALV.

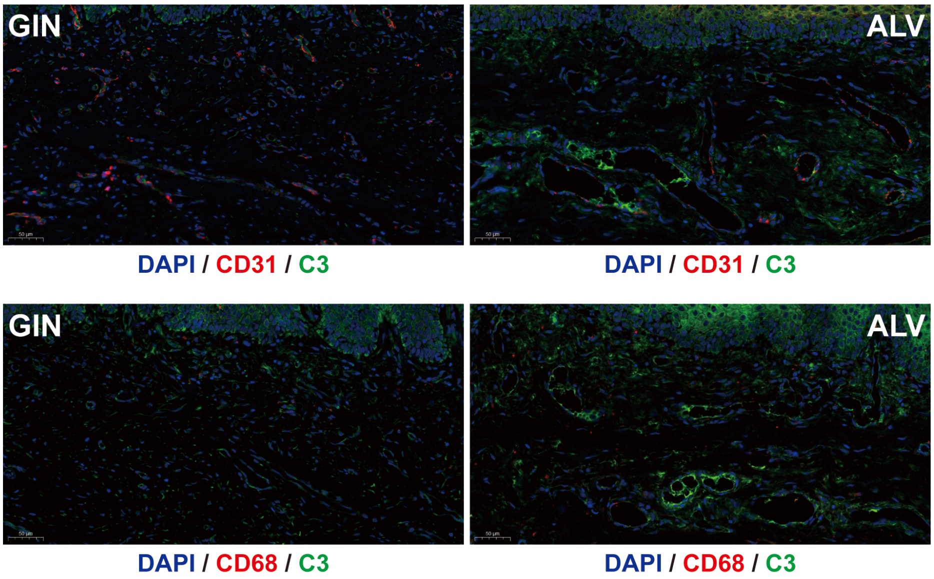

Supplementary Figure 6. Multiplex immunofluorescence co-staining of C3 with CD31 and CD68 in GIN and ALV.

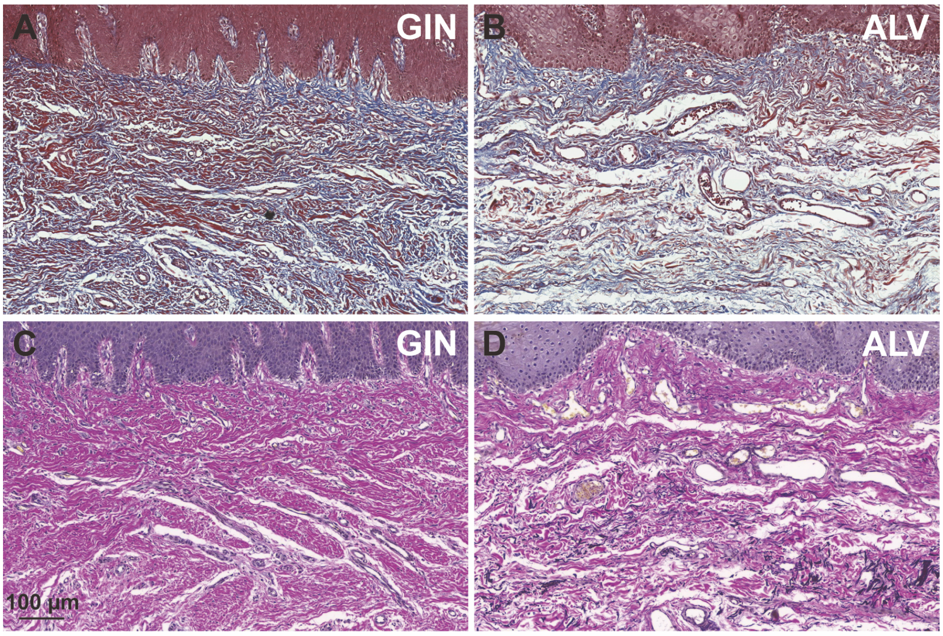

Supplementary Figure 7. Representative images of Masson's staining (A,B) and Elastic Van Gieson staining (C,D) for GIN and ALV.

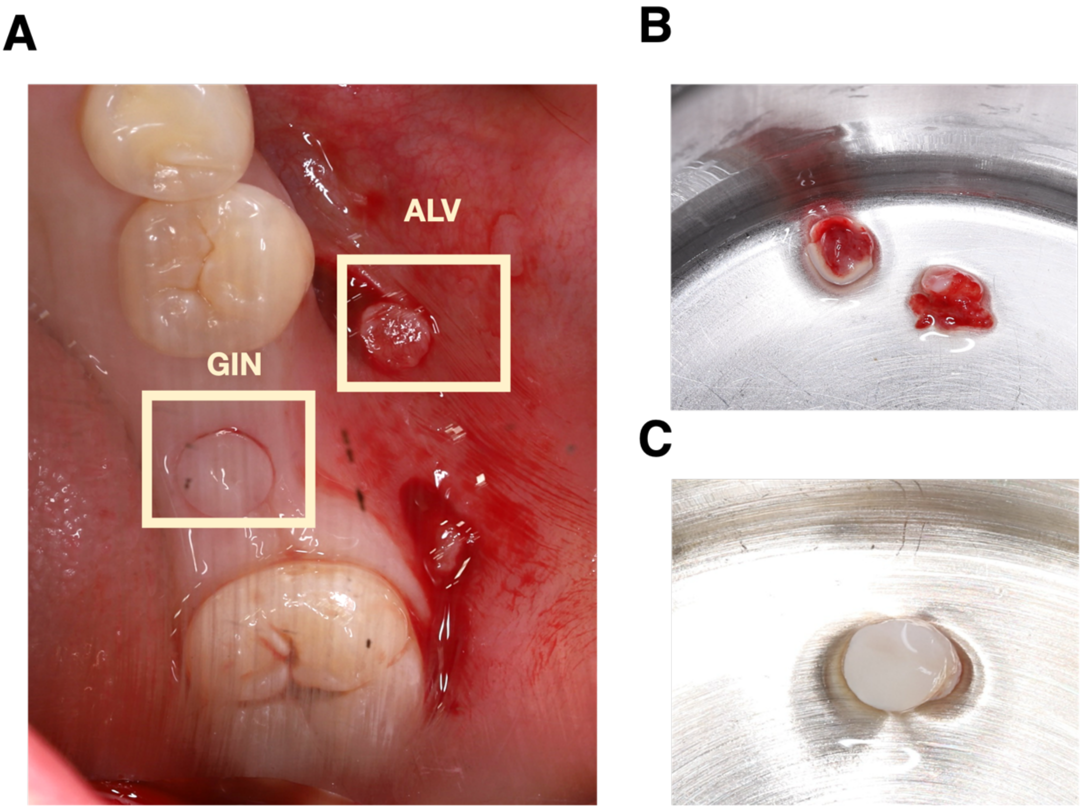

Supplementary Figure 8. Representative images of tissue sample collection. (A) Sampling sites for GIN and ALV (B) Representative image of keratinized gingiva (C) Representative image of non-keratinized gingiva.

Genes	Primer sequences
GAPDH	Forward 5'-ACATCGCTCAGACACCATG-3'
	Reverse 5'-TGTAGTTGAGGTCAATGAAGGG-3'
COL1A1	Forward 5'-CCCCTGGAAAGAATGGAGATG-3'
	Reverse 5'-CATCCAAACCACTGAAACCTC-3'
COL1A2	Forward 5'-AGGACAAGAAACACGTCTGG-3'
	Reverse 5'-GGTGATGTTCTGAGAGGCATAG-3'
COL3A1	Forward 5'-AAGTCAAGGAGAAAGTGGTCG-3'
	Reverse 5'-CTCGTTCTCCATTCTTACCAGG-3'
COL5A1	Forward 5'-TCGCTTACAGAGTCACCAAAG-3'
	Reverse 5'-GTTGTAGATGGAGACCAGGAAG-3'
COL5A2	Forward 5'-AGCAAACCCATCCAGTGTAC-3'
	Reverse 5'-TGGCTGTATTAGGTGATTGGTG-3'
COL6A1	Forward 5'-CGAATGCGAGATTTTGGACATC-3'
	Reverse 5'-ACGAAGTCCTTGGCAATCTC-3'
COL6A2	Forward 5'-TGAAACACGAAGCCTACGG-3'
	Reverse 5'-TCTCCCTGTCTTCCCTTCTG-3'
COL6A3	Forward 5'-CATTGGCTCTCACTGAAACAG-3'
	Reverse 5'-CCACAACCTCCATACCAGAATC-3'
COL6A6	Forward 5'-GGTTTTAAAGGCAGTGAAGGC-3'
	Reverse 5'-CTCCTGTCCATTTAGTCCTCTG-3'
COL12A1	Forward 5'-TGGTCGTGTGCAGAAATATAGG-3'
	Reverse 5'-CGGTGATAGTGTAAGGAGTGTC-3'
COL16A1	Forward 5'-GTGACCCCTGTGAAGTGTG-3'
	Reverse 5'-TCTCCTTTGATGCCTTGGATG-3'
TIMP3	Forward 5'-TGATGGCAAGATGTACACGG-3'
	Reverse 5'-GAAGTCACAAAGCAAGGCAG-3'
SERPINH1	Forward 5'-CCCTTCATCTTCCTAGTGCG-3'
	Reverse 5'-GTCTCAGGAGCCTTTGGATG-3'
PCOLCE	Forward 5'-GACACCTACTGCCGCTATG-3'
	Reverse 5'-GAGATCTGAGACGAACTGGAC-3'
FMOD	Forward 5'-CTCTCACACGTTCTCCAACC-3'
	Reverse 5'-GTCATCTTCATACTGGGCCTG-3'
ADAMTS2	Forward 5'-TGCACCTGGCAAGCATTGTT-3'
	Reverse 5'-GAGCCAAACGGACTCCAAGC-3'
MMP1	Forward 5'-GCACAAATCCCTTCTACCCG-3'
	Reverse 5'-TGAACAGCCCAGTACTTATTCC-3'
MFAP4	Forward 5'-TGTACCTCATCTACCCCTCG-3'
	Reverse 5'-CCCAGCTTGTAGTCATTCCAG-3'
ELN	Forward 5'-CCTGGCTTCGGATTGTCTC-3'
	Reverse 5'-CAAAGGGTTTACATTCTCCACC-3'
ADH1	Forward 5'-AGGATTTGACCTGCTTCACTC-3'
	Reverse 5'-TCCAGATCATGTAGGGTAGAGG-3'
ADH4	Forward 5'-GCATTGAAGAGGTTGAAGTAGC-3'
	Reverse 5'-AACGATCACTGGGAAAGCTAG-3'
ALDH1A2	Forward 5'-AAGATGTCTGGAAATGGGAGAG-3'
	Reverse 5'-GGGATCTTTACTGTCACCGTC-3'
ALDH1A3	Forward 5'-CTTCTGCCTTAGAGTCTGGAAC-3'
	Reverse 5'-CGTATTCACCTAGTTCTCTGCC-3'
AOX1	Forward 5'-GTCGATCCTGAAACAATGCTG-3'
	Reverse 5'-GGTGATGCCTTATCCTCTTGG-3'
CYP2C18	Forward 5'-AATCTCAGGTTGACCCAAAGG-3'
	Reverse 5'-AGCCAAACTATCTGCCCTTC-3'
CYP3A5	Forward 5'-AAGGAAGACTCACAGAACACAG-3'
	Reverse 5'-AAAGTCCATGTGTACGGGTC-3'
CYP26B1	Forward 5'-CCTCAGCGTCAAGTTCTTTG-3'
	Reverse 5'-GTTTCTACCTCCCACAACCAC-3'

Supplementary Table 1. Primer sequences for RT-qPC
